# Genome Sequence and Description of Paracoccus denitrificans Strain R-1, Isolated from Activated Sludge

**DOI:** 10.1128/mra.01236-21

**Published:** 2022-03-28

**Authors:** Chunchen Hu, Jiapeng Wu, Limei Wang, Yiguo Hong

**Affiliations:** a Institute of Environmental Research at Greater Bay Area, Key Laboratory for Water Quality and Conservation of the Pearl River Delta, Ministry of Education, Guangzhou University, Guangzhou, People’s Republic of China; Indiana University, Bloomington

## Abstract

Paracoccus denitrificans strain R-1 was isolated from an activated sludge sample from a sewage treatment plant in Taiwan. The complete genome, which was sequenced on the NovaSeq 6000 and PacBio Sequel platforms, consists of one chromosome with 4.05 Mb and one plasmid with 689 kb. Genome annotation predicts 4,167 protein-coding genes, 49 tRNAs, and 8 rRNAs.

## ANNOUNCEMENT

Paracoccus denitrificans is a coccoid, Gram-negative bacterium (1). A significant feature of this bacterium is its ability to convert nitrate to dinitrogen by denitrification (1). It is very important to further understand the genomic evolution of this taxon.

Paracoccus denitrificans strain R-1 was isolated from an activated sludge sample collected from Xinfeng Sewage Treatment Plant in Taiwan. Through 16S rRNA sequencing, the strain was identified as Paracoccus denitrificans. Genomic DNA was isolated using the HiPure soil DNA kit (Magen, China). Whole-genome sequencing was then performed on the Illumina NovaSeq platform and the PacBio Sequel II platform.

For the NovaSeq sequencing library, 200 μg genomic DNA was randomly fragmented by Ultrasonic breaker (Covaris S220) to an average size of 300 to 350 bp. The fragments were end repaired to add adaptors to both ends. The PCR products of each sample were cleaned and validated using an Agilent 2100 Bioanalyzer. The qualified libraries were sequenced (150-bp paired-end sequencing) on the NovaSeq system. Cutadapt v1.9.1 was used to process the raw data as follows: (i) remove primers and linker sequences; (ii) remove bases with mass values of <20 at both ends; (iii) remove sequences with reads lengths of <75 bp and with N base contents of >10%. The average read length is 149.49 bp, and the total number of reads is 19,275,562 after processing.

For the PacBio sequencing library, 5 to 10 μg genomic DNA was sheared into 10- to 15-kb fragments. A library was constructed using the SMRTbell Express template preparation kit v2.0. Sheared fragments were subjected to single-strand overhang removal, DNA damage repair, end repair, A-tailing, and barcoded overhang adapter ligation. Subsequent steps were performed according to the manufacturer’s instructions to prepare the SMRTbell library. The *N*_50_ value is 7,364 bp. A total of 40,522 sequences were acquired from single-molecule real-time (SMRT) long-read sequencing.

The final assembly results were obtained after error correction using Pilon v1.16. The coverage for the assembled genome is 99.99%. Two contigs were generated from the consensus assembly, i.e., one chromosome with 4,187,172 bp and a mean G+C content of 66.89% and one plasmid with 694,054 bp. The genome revealed 49 tRNA genes, 8 rRNA genes, and 23 noncoding RNA genes.

The predicted protein sequences encoded by the genes were compared with the protein sequences in the Gene Ontology (GO), KEGG, Clusters of Orthologous Genes (COG), NCBI nonredundant, CAZy, Pfam, and Swiss-Prot databases (2–6) using BLAST v2.2.31+ (7), and the genes with high levels of similarity were predicted to have similar functions as the proteins in the databases. A total of 4,167 protein-coding genes were predicted; 1,732 genes were potentially associated with metabolism. The predicted enzymes related to carbohydrate degradation, synthesis, and modification were mainly glycoside hydrolases and glycosyltransferases. Default parameters were used for the aforementioned software except where otherwise noted.

Gene annotation results would be conducive to understanding the metabolic mechanisms of *Paracoccus* species and to assessing the positive and negative effects of the organisms in the environment.

### Data availability.

The genome sequence was deposited in GenBank under the accession numbers CP087986, CP087987, and CP087988.
